# Bayesian Equivalence Testing and Meta-Analysis in Two-Arm Trials with Binary Data

**DOI:** 10.1155/2018/8134132

**Published:** 2018-08-08

**Authors:** Cynthia Kpekpena, Saman Muthukumarana

**Affiliations:** Department of Statistics, University of Manitoba, Winnipeg, MB, Canada R3T 2N2

## Abstract

We consider a Bayesian approach for assessing hypotheses of equivalence in two-arm trials with binary Data. We discuss the development of likelihood, the prior, and the posterior distributions of parameters of interest. We then examine the suitability of a normal approximation to the posterior distribution obtained via a Taylor series expansion. The Bayesian inference is carried out using Markov Chain Monte Carlo (MCMC) methods. We illustrate the methods using actual data arising from two-arm clinical trials on preventing mortality after myocardial infarction.

## 1. Introduction

Consider a clinical trial where a pharmaceutical company wants to test a new drug against a currently existing drug. Sometimes in these studies, the clinical trial end point may be the success or failure of the treatment. A binary outcome is an outcome whose unit can take on only two possible states “0” and “1.” This success/failure response variable could be heart disease (Yes/No), patient condition (Good/Critical), how often patient feel depressed (Never/Often), and so on. The natural distribution for modeling these types of binary data is the binomial distribution given by

(1) $$\begin{array}{c} f\left(x;p\right)=\left(\begin{array}{c} n\\ x \end{array}\right)p^{x}{\left(1-p\right)}^{n-x}\;\text{for }x=0,1,\ldots ,n,\;p\in \left(0,1\right). \end{array}$$

The mean and variance for the binomial random variable are *E*(*X*)=*np* and var(*X*)=*np*(1 − *p*), respectively. In (1), it is assumed that there are only two outcomes (denoted “success” or “failure”) and a fixed number of trials (*n*). The trials are independent with a constant probability of success.

The main objective of this type of clinical trial is to determine whether there is a significant difference between active treatment (new drug) and reference treatment (current drug). Tests of significance have generally been argued not to be enough. That is, if the *p* value for a test of significance leads to the nonrejection of the null hypothesis, it is not a proof that the null hypothesis holds. The clinician may want to test a null hypothesis of equivalence against an alternative hypothesis that states that there is a sufficient difference between the two drugs.

Equivalence testing is widely used when a choice is to be made between a drug (or a treatment) and an alternative. The term equivalence in the statistical sense is used to mean a weak pattern displayed by the data under study regarding the underlying population distribution. Equivalence tests are designed to show the nonexistence of a relevant difference between two treatments. It is known that Fisher's one-sided exact test is the same as the test for equivalence in the frequentist approach [1]. This testing procedure is similar to the classical two-sided test procedure but involves an equivalence zone determined by a margin known as equivalence margin (*δ*).

The equivalence margin (*δ*), which represents a margin of clinical indifference, is usually estimated from previous studies and as such is also based primarily on clinical criteria as well as statistical principle. This margin is influenced by statistical principle but largely dependent on the interest of the experimenter and research questions clinicians wish to answer. As such, the statistical method employed together with the design of the study must be in such a manner that the margin of difference is not too restrictive to capture the bounds of the research question. For a test of equivalence of two binomial proportions, the equivalence margin is discussed in [2].

The frequentist approach to equivalence testing is the two one-sided test (TOST) procedure. By the TOST, equivalence is established at the *α* significance level if a (1 − 2*α*) × 100% confidence interval for the difference in treatment means *μ*_*i*_ − *μ*_*j*_ is contained within the interval (−*δ*, *δ*) where *δ* is the equivalence margin.

The motivation for this paper is based on the fact that for a given disease, there is likely to be many other substitute drugs or new drugs that can be used to treat the patients. But these drugs may not all be at the same cost; some may possibly have adverse side effects, and the method of application could be complex for others. On grounds of these information, we do equivalence testing to see if two different drugs can be regarded as equivalent in terms of their treatment effect. There are a variety of different approaches to this problem as indicated by some recent literature. See Wellek [1], Albert [2], Gamalo et al. [3], Rahardja and Zhao [4] and Zaslavsky [5] for comprehensive details on recent developments. We remark that Gamalo et al. [3] consider a Bayesian approach to proportions along with noninferiority trials. In this paper, we consider a Bayesian approach focusing on equivalence tests. We also construct a simple normal approximation and provide a mechanism for missing data analysis as well.

The remaining sections of this article are organized as follows: In Section 2, Bayesian inferential procedure for binary data is discussed.  Section 3 presents a normal approximation to the posterior distribution obtained via a Taylor series expansion. We then examine the suitability of this normal approximation. We discuss a Gibbs sampling mechanism for estimating missing data in Section 4. In Section 5, we analyze a published dataset by Carlin [6] and Yusuf et al. [7]. This dataset consists with 22 treatment-control trials to prevent mortality after myocardial infarction. We conclude with a discussion of the approach in Section 5.

## 2. Bayesian Inferential Procedure

Let *X*_t_ be the number of individuals with positive exposure out of a total of *n*_t_ patients in treatment group with proportion *P*_t_. Accordingly, let *X*_c_ denote the number of individuals with positive exposure out of a total *n*_c_ in the control group with proportion *P*_c_. Then,

(2) $$\begin{array}{c} X_{t}∼\text{Bin}\left(n_{t},P_{t}\right),\\ X_{c}∼\text{Bin}\left(n_{c},P_{c}\right). \end{array}$$

The priors on the parameters, *P*_t_ and *P*_c_ are given by

(3) $$\begin{array}{c} P_{t}∼\text{Beta}\left(\alpha ,\beta \right),\\ P_{c}∼\text{Beta}\left(\varepsilon ,\eta \right). \end{array}$$

Then the posterior distributions of *P*_t_ and *P*_c_ are given by

(4) $$\begin{array}{c} \pi \left(P_{t}\;|\;X_{t}\right)\propto \text{Beta}\left(x_{t}+\alpha ,n_{t}+\beta -x_{t}\right),\\ \pi \left(P_{c}\;|\;X_{c}\right)\propto \text{Beta}\left(x_{c}+\varepsilon ,n_{c}+\eta -x_{c}\right). \end{array}$$

For Bayesian inference about treatment effect, a test is required to determine whether the posterior probability of treatment proportions *P*_t_ and *P*_c_ lies within the bounds of the equivalence margin or not. There is therefore, the need to sample from the posterior distribution of *P*_t_ − *P*_c_. The marginal posteriors of *P*_t_ and *P*_c_ are Beta distributions, and therefore *π*(*P*_t_ − *P*_c_ | *X*_t_, *X*_c_) is not in an analytically tractable form. So, *P*_1t_, *P*_2t_,…, *P*_*n*t_ are generated from *π*(*P*_t_ | *X*_t_) and independently *P*_1c_, *P*_2c_,…, *P*_*n*c_ generated from *π*(*P*_c_ | *X*_c_) because *P*_t_ and *P*_c_ are independent. Then, *P*_1t_ − *P*_1c_, *P*_2t_ − *P*_2c_,…, *P*_*n*t_ − *P*_*n*c_ can be treated as a random sample from *π*(*P*_t_ − *P*_c_ | *X*_t_, *X*_c_).

## 3. Normal Approximation to the Beta Posterior Distribution

Note that the posterior distributions of *P*_t_ and *P*_c_ are Beta distributions. By following Kpekpena [8], a normal approximation to posteriors can be obtained using a Taylor series expansion of the Beta distribution. By applying a Taylor series expansion with first three terms, it can be shown that *π*(*P*_t_ | *X*_t_) ≈ *𝒩*(*μ*, *σ*), where

(5) $$\begin{array}{c} \mu =\frac{1-\alpha -x_{t}}{2-\alpha -n_{t}-\beta },\\ \sigma =\frac{1}{{\left[-{\left(2-\alpha -n_{t}-\beta \right)}^{3}/\left(\left(1-x_{t}-\alpha \right)\left(1-n_{t}-\beta +x_{t}\right)\right)\right]}^{1/2}}. \end{array}$$

**Figure 1:**
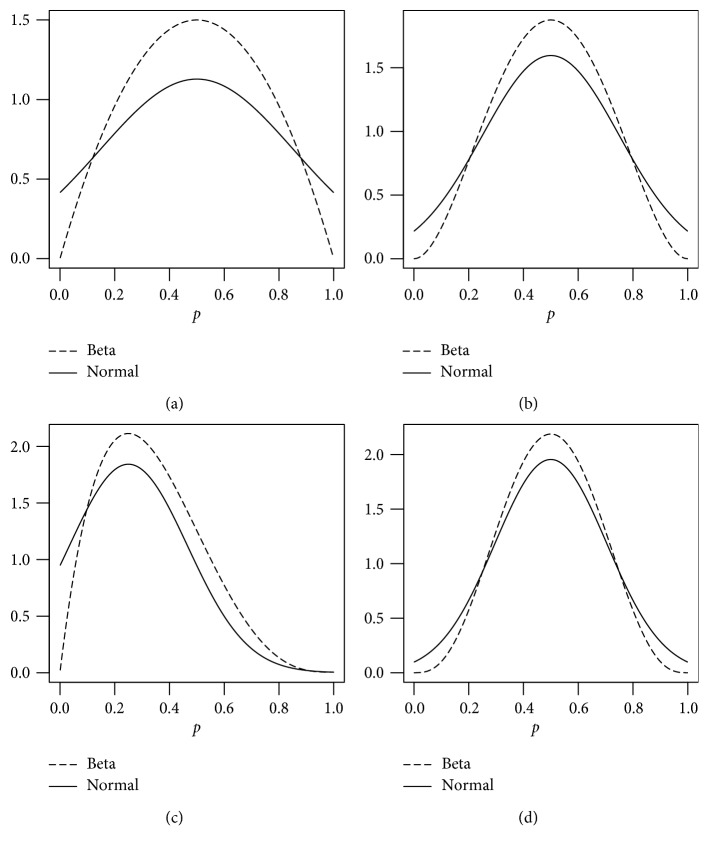
The normal approximations of (a) Beta(2, 2), (b) Beta(3, 3), (c) Beta(2, 4), and (d) Beta(4, 4).

**Figure 2:**
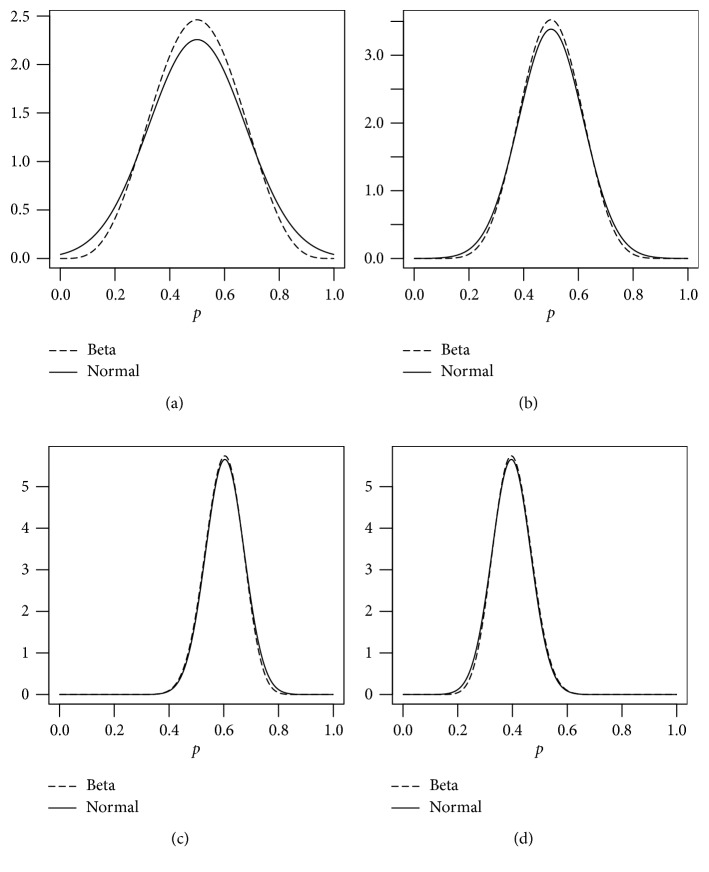
The Normal approximations (a) Beta(5, 5), (b) Beta(10, 10), (c) Beta(30, 20), and (d) Beta(20, 30).

Similarly, the approximation of *π*(*P*_c_ | *X*_c_) can also be obtained. The details of this construction are given in the Appendix. We provide some approximations based on this development in Table 1 and Figures 1 and 2. It is clear that the approximation starts to work well for the values of the posterior parameters from *x*+*α*=10 and *n*+*β* − *x*=10. However, the approximation is not suitable when Beta posterior parameters are less than 10.

## 4. Estimating Missing Data in Arms

Missing data are easily handled in Bayesian inference by treating them as another set of parameters. We estimate the missing values conditioning on the observed data. For example, let *X*_1_,…, *X*_*n*_ be a binary random sample from Ber(*P*) in an arm and suppose that *X*_*m*_ is missing. Note that *P* represents *P*_t_ in treatment arm and *P*_c_ in control group. Let *P* ~ Beta(*α*, *β*) and *Y*=∑_*i*≠*m*_^*n*^ *X*_*i*_. Then, the likelihood of the observed data is

(6) $$\begin{array}{c} L\left(X_{\text{obs}}\;|\;P\right)=\left(\begin{array}{c} n-1\\ y \end{array}\right)P^{y}{\left(1-P\right)}^{n-1-y}. \end{array}$$

The posterior of *P* based on the complete data *X*=(*Y*, *X*_*m*_) is

(7) $$\begin{array}{c} \pi \left(P\;|\;X\right)\propto P^{y+x_{m}}{\left(1-P\right)}^{n-y-x_{m}}\frac{1}{B\left(\alpha ,\beta \right)}P^{\alpha -1}{\left(1-P\right)}^{\beta -1}. \end{array}$$

The full conditionals of *P* and *X*_*m*_ are

(8) $$\begin{array}{c} \pi \left(P\;|\;y,x_{m}\right)∼\text{Beta}\left(y+x_{m}+\alpha ,n-y-x_{m}+\beta \right),\\ \pi \left(x_{m}\;|\;y,P\right)∼\text{Ber}\left(P\right). \end{array}$$

It is easy to generate from these full conditionals in *R*, so *P* and *x*_*m*_ can be estimated using Gibbs sampling.

## 5. Data Analysis

We apply our approach on data analyzed in [7, 9]. The data includes 22 trials of beta-blockers to prevent mortality after myocardial infarction. For each of the 22 trials, a test of equivalence is done to ascertain whether the treatment proportion is equivalent to the control proportion. This example is based on the Statistical inferential procedure for binary data discussed in Sections 2 and 3. For each arm, the number of patients who had myocardial infarction out of a total *n*_t_ is considered to be the number of successes in *n*_t_ binomial trials. Similarly, the number of cases in the control group is treated as a binomial outcome independent of the treatment group. The equivalence margin *δ* is chosen to be as small as possible such that if the absolute value of the difference in the control and treatment proportions is less than *δ*, and we can say that the two proportions are equivalent. For demonstration purpose, we assume a practically meaningful equivalence margin *δ*=0.01. We use noninformative priors Beta(2, 1) for the parameters, *P*_t_ and *P*_c_. The hypothesis for a test of equivalence of study number *i* and its control group is as follows:

(9) $$\begin{array}{c} H_{0}:\left|P_{ti}-P_{ci}\right|\geq \delta ,\\ H_{1}:\left|P_{ti}-P_{ci}\right|<\delta . \end{array}$$

We perform the equivalence test in (9) using the Bayes factor [9].  Table 2 gives the results of the equivalence tests. The first column *D*_*i*_ is the *i*th study label. Columns 2 and 3 are the treatment proportion (*x*_t_/*n*_t_) and control proportion (*x*_c_/*n*_c_), respectively. Columns 4 (*P*(*H*_1_ | *X*)) and 6 (*P*_*A*_(*H*_1_ | *X*)) are the posterior probabilities that *H*_1_ : |*P*_t*i*_ − *P*_c*i*_| < *δ* is true under the Beta posterior distributions and under the normal approximation to the Beta posterior, respectively. Column 5 (*B*) is the Bayes factor for exact posterior, and *B*_*A*_ in column 7 is the Bayes Factor based on the normal approximation. For study 1, the Bayes factor for the exact posterior is 7.3822 whereas that of the normal approximation is 7.5466. Both Bayes factors are above 1 which implies that *H*_0_ is more likely to be true, and *H*_0_ is the hypothesis that the treatment proportion is not equivalent to the control proportion. We remark that classical hypothesis tests give one hypothesis a preferred status and only consider evidence against it which is not the case in Bayesian tests. Results also indicate that the approximation and exact computation lead to the same conclusion in each study indicating the suitability of the approximation.

**Figure 3:**
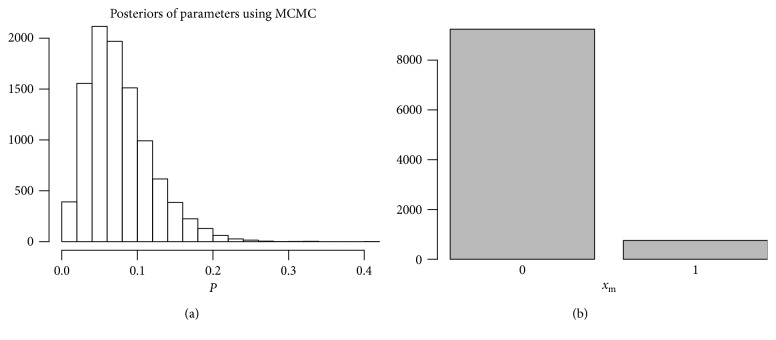
The distribution of *x*_*m*_ shows it is more likely to be 0.

**Figure 4:**
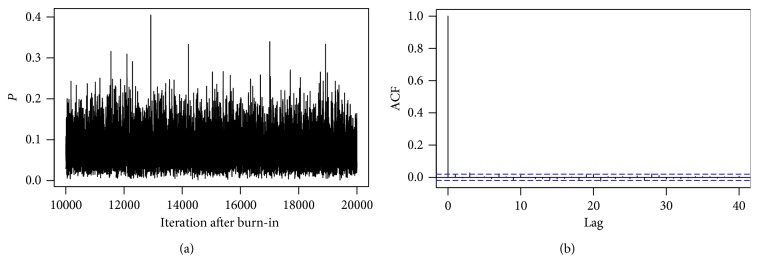
There is no discernible pattern in the trace plot and no large spikes in the autocorrelation plot.

We now consider a missing data analysis in an arm. As an example, suppose an observation was missing in the treatment group under study 1. We estimate this missing value using Gibbs sampling derived in Section 4. The posterior distributions of parameters *P* and *x*_*m*_ are given in Figure 3 based on 20000 MCMC simulations. According to Figure 3, it is likely that *x*_*m*_ is 0. The trace plot in Figure 4 shows that mixing is good enough, and there are no large spikes in the autocorrelation plot after lag 0. This is an indication of convergence of the Markov Chain.

We also consider a meta-analysis of the binary data in two-arm trials in order to assess the between-study variations. Let *y*_*i*_ be the estimate of the true effect size *μ*_*i*_ corresponding to the *i*th study. Then, the random effects model is given as

(10) $$\begin{array}{c} y_{i}=\mu_{i}+e_{i}\;\text{where }e_{i}∼N\left(0,\sigma_{i}^{2}\right). \end{array}$$

As developed in Muthukumarana and Tiwari [10], we consider a hierarchical Dirichlet process formulation for *μ*_*i*_ as follows:

(11) $$\begin{array}{c} \mu_{i}\;|\;F∼F,\\ F∼\text{DP}\left(M,H\right),\\ H∼N\left(\mu ,\tau^{2}\right),\\ \mu ∼N\left(\mu_{0},d\tau^{2}\right),\\ \frac{1}{\tau^{2}}∼G\left(a,b\right), \end{array}$$

where *M*, *μ*_0_, and *d* are known.

We analyze the dataset published in Nissen and Wolski [11] to assess the between-study heterogeneity. In this dataset, there are 42 trials including 15565 diabetes patients who were put on rosiglitazone (treatment group) and 12282 diabetes patients assigned to medication that does not contain rosiglitazone (control group). Note that the interest is on myocardial infarction and death from rosiglitazone as a treatment for diabetes. We use the odds ratio as the treatment effect. The parameters of the model are estimated by Gibbs sampling algorithm implemented in R. The estimates of the model parameters (*μ*_*i*_) are given in Table 3.

We also conducted a simulation study to assess the validity of the approach. Each study was simulated by means of a binomial random variable in which the number of cases in the treatment group and the control group are generated as independent binomial random variables. We generate twenty binomial successes using the rbinom random generator. We assume *n*=200 in each case and fix the *p* at 0.7. This setting is similar to administering a treatment in twenty hospitals with 200 patients in each hospital. Fixing *p* at 0.7 generates number of cases that do not vary so much from each other. This is confirmed in the non-significance of the chi-square test for heterogeneity. Another set of twenty “number of cases” is generated from the binomial distribution, but this time we induce heterogeneity. This is done by varying the success probability of each trial. For instance rbinom(1, 200, 0.86), rbinom(1, 200, 0.10), rbinom(1, 200, 0.55),….

Note that our interest is in comparing the posterior treatment means of the heterogeneous studies with the studies that are not heterogeneous.  Table 4 compares the posterior treatment means of 20 studies with heterogeneity to the treatment means of 20 other studies in which there is no heterogeneity. Column 1 is the posterior treatment means of the nonheterogeneous (*μ*_*i*_) studies whereas *μ*_*i*_^*∗*^ in column 2 is the posterior treatments of the heterogeneous studies. Treatment means in column 1 (*μ*_*i*_) are mostly 0.68 or just slightly below or above it. If the responses are similar, the treatment effects are supposed to be an estimate of a common treatment mean. On the other hand, all the treatment means in column 2 (*μ*_*i*_^*∗*^) differ from each other significantly indicating that the model can assess the between-study variations.

## 6. Discussion

We have considered a Bayesian analysis of binary data in testing hypotheses of equivalence. The tests of hypotheses of equivalence are popular in clinical trials, and our approach is relatively simple and easy to perform. Bayesian formulation was considered for testing hypothesis of equivalence, and we observed that the normal approximation to the Beta posterior can be used for moderately large sample sizes. We also presented a mechanism for estimating missing data in arms. This is useful in situations where data are partially missing in some arms. We also considered a meta-analytic approach for assessing between-study variations.

There are two directions we would like to pursue along the methods discussed in this article. We are interested in enhancing the method to accommodate extra covariates into the model in the presence of multiple outcomes in an arm. The incorporation of covariates makes the Bayes factor inappropriate, and we would like to examine the other model selection criterion in place of the Bayes factor.

## Figures and Tables

**Table 1:** Normal approximation to the Beta distribution.

Exact distribution	Approximation
Beta(2, 1)	*N*(1, *∞*)
Beta(1, 2)	*N*(0, *∞*)
Beta(10, 10)	*N*(0.5000, 8.4853)
Beta(5, 1)	*N*(1, *∞*)
Beta(1, 5)	*N*(0, *∞*)
Beta(2, 2)	*N*(0.5000, 2.8284)
Beta(3, 3)	*N*(0.5000, 4.0)
Beta(2, 4)	*N*(0.2500, 4.6188)
Beta(4, 4)	*N*(0.5000, 4.8990)
Beta(5, 5)	*N*(1, 5.6569)
Beta(30, 20)	*N*(0.6042, 14.1673)
Beta(20, 30)	*N*(0.3958, 14.1673)
Beta(50, 20)	*N*(0.7206, 18.3776)
Beta(20, 50)	*N*(0.2794, 18.3776)

**Table 2:** Posterior probabilities and Bayes factor.

*D*_*i*_	*P*_t*i*_	*P*_c*i*_	*P*(*H*_1_ | *X*)	*B*	*P*_*A*_(*H*_1_ | *X*)	*B*_*A*_
*D*_1_	0.0789	0.0769	0.1193	7.3822	0.1170	7.5466
*D*_2_	0.0614	0.1207	0.0725	12.7931	0.0669	13.9506
*D*_3_	0.0725	0.1183	0.1184	7.4459	0.1132	7.8283
*D*_4_	0.0665	0.0836	0.2343	3.2680	0.2294	3.3583
*D*_5_	0.0789	0.0740	0.3718	1.6896	0.3710	1.6957
*D*_6_	0.0678	0.1154	0.0998	9.02004	0.0962	9.3964
*D*_7_	0.1037	0.1618	0.0015	665.6667	0.0010	971.6452
*D*_8_	0.0949	0.1019	0.3861	1.5900	0.3854	1.5949
*D*_9_	0.0899	0.1312	0.0787	11.7064	0.0784	11.7538
*D*_10_	0.0720	0.0979	0.0432	22.1482	0.0395	24.3175
*D*_11_	0.0733	0.0892	0.2950	2.3898	0.2958	2.3802
*D*_12_	0.1711	0.1767	0.2317	3.3159	0.2341	3.2722
*D*_13_	0.0309	0.0546	0.1951	4.1256	0.1900	4.2626
*D*_14_	0.0664	0.0510	0.3030	2.3003	0.3022	2.3089
*D*_15_	0.1623	0.2109	0.0989	9.1112	0.0994	9.0554
*D*_16_	0.1594	0.1784	0.1871	4.3447	0.1886	4.3010
*D*_17_	0.1116	0.0984	0.2193	3.5560	0.2214	3.5147
*D*_18_	0.0530	0.0390	0.2673	2.7411	0.2649	2.7754
*D*_19_	0.0345	0.0224	0.3175	2.1496	0.3293	2.0372
*D*_20_	0.1531	0.1835	0.1584	5.3131	0.1552	5.4439
*D*_21_	0.0691	0.1181	0.0331	29.2110	0.0311	31.1380
*D*_22_	0.0324	0.0579	0.0932	9.7296	0.0867	10.536

**Table 3:** The estimates of posterior treatments and standard deviations.

Parameter	Estimate	Standard deviation
*τ* ^2^	0.74	0.2794073
*μ*	0.71	0.4142608
*μ*_1_	−0.73	0.8151172
*μ*_2_	−0.63	0.7358434
*μ*_3_	1.2	0.4914719
*μ*_4_	−1.1	0.6541732
*μ*_5_	−0.74	0.8379231
*μ*_6_	−0.77	0.7702218
*μ*_7_	−0.70	0.8191522
*μ*_8_	−0.61	0.7714286
*μ*_9_	−0.74	0.7989194
*μ*_10_	−0.75	0.8194161
*μ*_11_	−0.77	0.7766973
*μ*_12_	−0.74	0.8008516
*μ*_13_	−0.67	0.7848413
*μ*_14_	−0.73	0.8148389
*μ*_15_	−0.65	0.7427869
*μ*_16_	−0.72	0.6874473
*μ*_17_	−0.67	0.6670385
*μ*_18_	−0.74	0.8121317
*μ*_19_	−0.69	0.7916802
*μ*_20_	−0.74	0.8151257
*μ*_21_	−0.78	0.7748052
*μ*_22_	−0.78	0.7449046
*μ*_23_	−0.71	0.8175963
*μ*_24_	−0.75	0.8185433
*μ*_25_	−1.6	0.4702446
*μ*_26_	−0.57	0.5950003
*μ*_27_	−0.74	0.8104195
*μ*_28_	−0.74	0.8192542
*μ*_29_	−0.74	0.8091605
*μ*_30_	−0.73	0.8372302
*μ*_31_	−0.58	0.6008941
*μ*_32_	−0.72	0.8096048
*μ*_33_	−0.76	0.8134446
*μ*_34_	−0.69	0.6464147
*μ*_35_	−0.72	0.8196591
*μ*_36_	−0.75	0.8109735
*μ*_37_	−0.71	0.8172117
*μ*_38_	−0.72	0.8127885
*μ*_39_	−0.74	0.8024024
*μ*_40_	−0.73	0.8114458
*μ*_41_	−0.189	0.5258747
*μ*_42_	0.01	0.3224310

**Table 4:** Estimates of treatment means for twenty studies with 200 observations within each study.

Study	*μ*_*i*_	*μ*_*i*_^*∗*^
1	0.68	0.092
2	0.67	0.39
3	0.67	0.80
4	0.68	0.69
5	0.68	0.76
6	0.68	−2.8
7	0.68	−1.4
8	0.68	0.35
9	0.68	−0.11
10	0.70	0.53
11	0.67	0.69
12	0.70	−0.054
13	0.68	0.72
14	0.67	0.39
15	0.68	0.41
16	0.69	0.79
17	0.67	0.81
18	0.69	−0.94
19	0.68	−1.6
20	0.69	0.81

## Data Availability

The data used to support the findings of this study are available from the corresponding author upon request.

## Appendix

Let the best estimate of *P*, *P*_0_ be the value of *P* for which the posterior is at its maximum. That is,

(A.1) $$\begin{array}{c} \frac{d\pi \left(P\;|\;x\right)}{dp}{\;|\;}_{P_{0}}=0,\\ \frac{d^{2}\pi \left(P\;|\;x\right)}{dP^{2}}{\;|\;}_{P_{0}}<0. \end{array}$$

The Taylor series expansion of a function *f*(*x*) at *X*=*x*_0_ is

(A.2) $$\begin{array}{c} f\left(x\right)=\overset{\infty }{\underset{m=0}{\sum }}\frac{f^{m}\left(x_{0}\right)}{m!}{\left(x-x_{0}\right)}^{m}. \end{array}$$

Let the log of the posterior distribution be

(A.3) $$\begin{array}{c} L\left(P\right)=\log\left(\pi \left(P\;|\;X\right)\right). \end{array}$$

By applying a Taylor series expansion to *L*(*P*) at *P*_0_ with first three terms,

(A.4) $$\begin{array}{c} L\left(P\right)=L\left(P_{0}\right)+\frac{dL\left(P\right)}{dP}{\;|\;}_{P_{0}}\left(P-P_{0}\right)+\frac{1}{2}\frac{d^{2}L\left(P\right)}{dP^{2}}{\;|\;}_{P_{0}}{\left(P-P_{0}\right)}^{2}+\cdots \\ =\text{constant}+\frac{1}{2}\frac{d^{2}L\left(P\right)}{dP^{2}}{\;|\;}_{P_{0}}{\left(P-P_{0}\right)}^{2}+\cdots . \end{array}$$

By taking the exponential of *L*(*P*),

(A.5) $$\begin{array}{c} \pi \left(P\;|\;X\right)\propto K\;{\exp^{\left(1/2\right)\left(d^{2}L\left(P\right)/dP^{2}\right)}\;|\;}_{P_{0}}{\left(P-P_{0}\right)}^{2}, \end{array}$$

where *K* is a normalizing constant.

Let *μ*=*P*_0_ and *σ*=1/[(−*d* ^2^ *L*(*P*))/*dP* ^2^∣_*P*_0__]^1/2^. This gives

(A.6) $$\begin{array}{c} \pi \left(P\;|\;X\right)\approx N\left(\mu ,\sigma \right),\\ \pi \left(P_{t}\;|\;X_{t}\right)∼\text{Beta}\left(x_{t}+\alpha ,n_{t}+\beta -x_{t}\right)\\ ∼P_{t}^{x_{t}+\alpha -1}{\left(1-P_{t}\right)}^{n_{t}+\beta -x_{t}-1}\\ \Rightarrow L\left(P\right)=k+\left(x_{t}+\alpha -1\right)\log P_{t}+\left(n_{t}+\beta -x_{t}-1\right)\log P_{t},\\ \frac{dL\left(P_{t}\right)}{dP_{t}}=\frac{\left(x_{t}+\alpha -1\right)}{P_{t}}-\frac{\left(n_{t}+\beta -x_{t}-1\right)}{1-P_{t}}=0\\ \Rightarrow \left(1-P_{t}\right)\left(x_{t}+\alpha -1\right)-P_{t}\left(n_{t}+\beta -x_{t}-1\right)=0,\\ \alpha -1+x_{t}+2P_{t}-\alpha P_{t}-n_{t}P_{t}-\beta P_{t}=0,\\ 2P_{t}+x_{t}+\alpha -1-\alpha P_{t}-n_{t}P_{t}-\beta P_{t}=0,\\ P_{0}=\frac{1-\alpha -x_{t}}{2-\alpha -n_{t}-\beta },\\ \frac{dL\left(P_{t}\right)}{dP_{t}}=\left(x_{t}+\alpha -1\right)P_{t}^{-1}-\left(n_{t}+\beta -x_{t}-1\right){\left(1-P_{t}\right)}^{-1},\\ \frac{d^{2}}{dP_{t}^{2}}\left(\pi \left(P_{t}\;|\;X_{t}\right)\right)=-\left(x_{t}+\alpha -1\right)P_{t}^{-2}-\left[-\left(-1\right){\left(1-P_{t}\right)}^{-2}\left(n_{t}+\beta -x_{t}-1\right)\right]\\ =\frac{-\left(x_{t}+\alpha -1\right)}{P_{t}^{2}}-\frac{-\left(n_{t}+\beta -x_{t}-1\right)}{{\left(1-P_{t}\right)}^{2}},\\ 1-P_{0}=1-\frac{1-\alpha -x_{t}}{2-\alpha -n_{t}-\beta }\\ =\frac{2-\alpha -n_{t}-\beta -1+x_{t}+\alpha }{2-\alpha -n_{t}-\beta }\\ =\frac{1-n_{t}-\beta +x_{t}}{2-\alpha -n_{t}-\beta },\\ \frac{d^{2}}{dP_{t}^{2}}{\left(\pi \left(P_{t}\;|\;X_{t}\right)\right)\;|\;}_{P_{0}}=\frac{\left(1-x_{t}-\alpha \right)}{{\left[\left(1-x_{t}-\alpha \right)/\left(2-\alpha -n_{t}-\beta \right)\right]}^{2}}+\frac{\left(1-n_{t}-\beta +x_{t}\right)}{{\left[\left(1-n_{t}-\beta +x_{t}\right)/\left(2-\alpha -n_{t}-\beta \right)\right]}^{2}}\\ =\left(1-x_{t}-\alpha \right)\times \frac{{\left(2-\alpha -n_{t}-\beta \right)}^{2}}{{\left(1-x_{t}-\alpha \right)}^{2}}+\left(1-n_{t}-\beta +x_{t}\right)\times \frac{{\left(2-\alpha -n_{t}-\beta \right)}^{2}}{{\left(1-n_{t}-\beta +x_{t}\right)}^{2}}\\ =\frac{{\left(2-\alpha -n_{t}-\beta \right)}^{2}}{1-x_{t}-\alpha }+\frac{{\left(2-\alpha -n_{t}-\beta \right)}^{2}}{1-n_{t}-\beta +x_{t}}\\ ={\left(2-\alpha -n_{t}-\beta \right)}^{2}\left[\frac{1-n_{t}-\beta +x_{t}+1-x_{t}-\alpha }{\left(1-x_{t}-\alpha \right)\left(1-n_{t}-\beta +x_{t}\right)}\right]\\ =\frac{{\left(2-\alpha -n_{t}-\beta \right)}^{3}}{\left(1-x_{t}-\alpha \right)\left(1-n_{t}-\beta +x_{t}\right)},\\ \sigma =\frac{1}{{\left[{-\left(d^{2}/dP_{t}^{2}\right)\left(\pi \left(P_{t}\;|\;X_{t}\right)\right)\;|\;}_{P_{0}}\right]}^{1/2}}\\ =\frac{1}{{\left[\left(-{\left(2-\alpha -n_{t}-\beta \right)}^{3}\right)/\left(\left(1-x_{t}-\alpha \right)\left(1-n_{t}-\beta +x_{t}\right)\right)\right]}^{1/2}}. \end{array}$$
